# Thirty years of nutrients and biogeochemistry in the Norwegian, Greenland and Iceland Seas, 1990–2019

**DOI:** 10.1038/s41597-023-02156-5

**Published:** 2023-05-06

**Authors:** Kjell Gundersen, Jane S. Møgster, Vidar Lien, Elizaveta Ershova, Linda F. Lunde, Hilde Arnesen, Ann-Kristin Olsen

**Affiliations:** 1 Plankton Research Group, Institute of Marine Research, P.O. Box 1870 Nordnes, NO-5817 Bergen, Norway; 2 Oceanography and Climate Group, Institute of Marine Research, P.O. Box 1870 Nordnes, NO-5817 Bergen, Norway

**Keywords:** Biogeochemistry, Chemical biology

## Abstract

This dataset contains biogeochemical samples analyzed by the Plankton Chemistry Laboratory at the Institute of Marine Research (IMR), from the Norwegian, Greenland and Iceland Seas. Number of surveys and stations have varied greatly over the last 3 decades. IMR is conducting one annual Ecosystem Survey in April-May each year, with multiple trawl surveys and net tows, but only CTD water collections are reported here. This month-long exercise also has companion vessels from Iceland and the Faroe Islands surveying their own territorial waters. Three transects are the core of the time-series, visited multiple times each year (Svinøy-NorthWest, Gimsøy-NorthWest, Bjørnøya-West). On each station, the CTD cast is sampled for dissolved inorganic nutrients (nitrate, nitrite, phosphate, silicate) and phytoplankton chlorophyll-*a* and phaeopigments (ChlA, Phaeo) at predetermined depths. At times, short-term projects have collected samples for Winkler dissolved oxygen titrations (DOW) and particulate organic carbon and nitrogen (POC, PN) determinations. This unique data set has seen limited use over the years but is a great contribution towards global ocean research and climate change investigations.

## Background & Summary

**Fig. 1:**
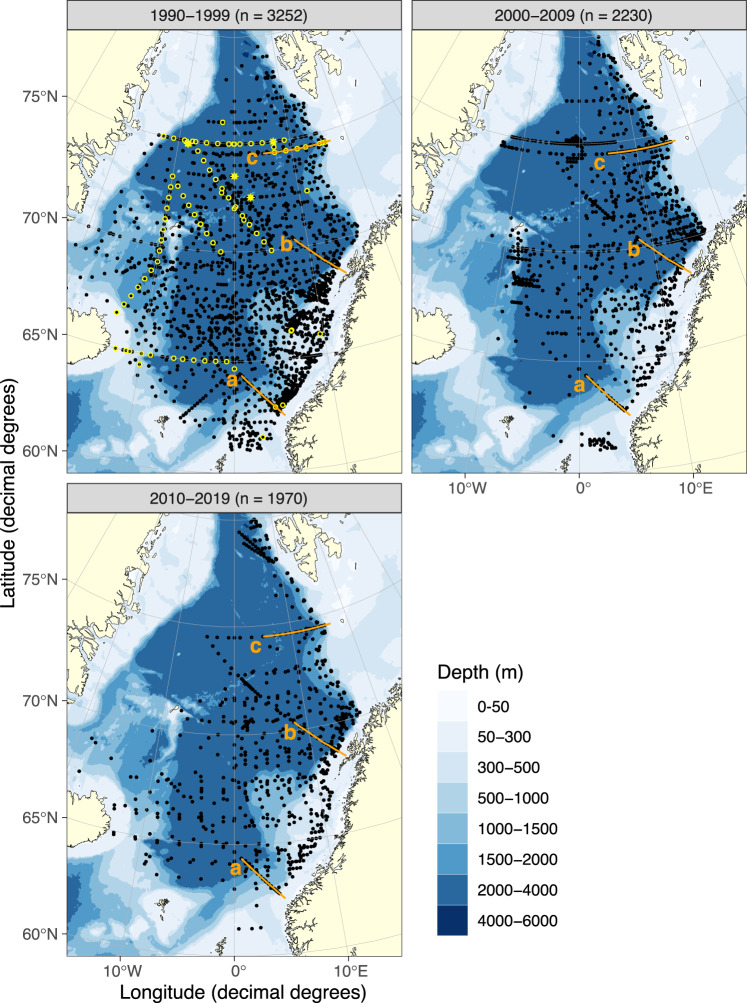
Distribution of sampling stations over three decades in the Norwegian, Greenland and Iceland Seas (black dots). The seasonal transects (orange lines) in the *Open Ocean Monitoring Survey*, are shown for Svinøy – NW (**a**), Fugløya – Bjørnøya (**b**) and Bjørnøya – W (**c**). Total number of stations visited (n) is summarized for each decade. Selected stations sampled for DOW (yellow circles) and POC, PN (yellow stars) in 1993, are also shown (profiles shown in Figs. 7, 8).

Major and extensive surveys in the Norwegian Sea region were more formalized in the *Mare cognitum* program^1^. This was a follow-up from the successful *PRO MARE* program (Norwegian Research Programme for Marine Arctic Ecology, 1984–1989) in the Barents Sea and the *MARE NOR* program (Norwegian Research Programme on North Norwegian Coastal Ecology, 1990–1994), both hosted by the IMR. *Mare cognitum* lasted almost a decade (1993–2001) as Norway’s contribution to the international *GLOBEC* program (Global Ocean Ecosystem Dynamics), and was collated by a number of additional, collaborative projects initiated at IMR and by other Norwegian and foreign research institutions. Cruises to the region were also supported in kind by the Marine Research Institute in Iceland and the Faroese Fisheries Laboratory at the Faroe Islands. At that time, a growing concern for climate change spurred a new-found interest for research into marine resource biology in the Nordic Seas. Initiation of the *Open Ocean Time-series Program* (Havovervåkingen) in the Norwegian Sea, was adopted from the continuing surveys in the Barents Sea. A number of transect lines (Snittundersøkelsene) have also been visited over the years and three transects (Svinøy-NorthWest, Gimsøy-NorthWest, and Bjørnøya-West) are still to this date surveyed multiple times each year (see Fig. 1 for details). The annual *Ecosystem Survey* is a separate, coordinated effort between Norway, Iceland, and the Faroe Islands, and IMR usually contribute with 2 research vessels for trawl surveys, net tow collections and CTD water sampling. Additionally, water samples are routinely collected from a number of short-term sampling projects by all major ships operated by the IMR in the region and throughout each year [see Gundersen *et al*.^2^ for an overview]. Each CTD station is sampled for dissolved inorganic nutrients (nitrate, nitrite, phosphate, silicate) and phytoplankton pigments (ChlA, Phaeo) at predetermined depths (Table 1), for later analysis at IMR. On occasion, short-term projects have also conducted particulate organic carbon and nitrogen (POC, PN) determinations and Winkler oxygen titrations (DOW). This set of quality-controlled data, from 30 years of ocean surveys conducted by the IMR, has never been published in its entirety.

**Table 1:** A guide to standardized sampling depth intervals of CTD stations at various depths (designated bottom line in table).

Water bottle number												
	1	2	3	4	5	6	7	8	9	10	11	12
Sample depths (m)	50	30	20	10	5							
	75	50	30	20	10	5						
	100	75	50	30	20	10	5					
	125	100	75	50	30	20	10	5				
	150	125	100	75	50	30	20	10	5			
	200	150	125	100	75	50	30	20	10	5		
	250	200	150	125	100	75	50	30	20	10	5	
	300	250	200	150	125	100	75	50	30	20	10	5
	400	300	250	200	150	100	75	50	30	20	10	5
	500	400	300	200	150	100	75	50	30	20	10	5
	600	500	400	300	200	150	100	75	50	30	10	5
	800	600	500	400	300	200	150	100	75	50	30	10
	1000	800	500	400	300	200	150	100	75	50	30	10
	1200	1000	800	500	400	300	200	150	100	50	30	10
	1500	1200	1000	800	500	400	300	200	100	50	30	10
	1800	1500	1200	1000	800	500	400	200	100	50	30	10
	2000	1800	1500	1000	800	500	400	200	100	50	30	10
	2500	2000	1500	1000	800	500	400	200	100	50	30	10
	3000*	2500*	2000*	1500*	1000*	800*	500*	200*	100*	50*	30*	10*

*)Nominal station depth (m).

Nutrient samples (nitrate, nitrite, phosphate, silicate) are collected from the maximum number of depths at each CTD station (12 depths), while the number of pigment samples (ChlA, Phaeo; grey area) are only collected in the upper 200 m of the water column. On dedicated cruises, Winkler DO (DOW), and particulate organic carbon and nitrogen (POC, PN) samples were collected more at random and with a focus on depths in the upper 600 m of the water column (specific depths are not shown here).

## Methods

### Sample collection and analysis

Seawater samples are collected from Niskin-type water bottles at predetermined depths (Table 1) triggered by a CTD (Conductivity Temperature Depth) unit mounted on a rosette. Two types of CTD have been used on our survey ships over the years (see Gundersen *et al*.^2^ for details) and total numbers of stations sampled has also varied over the years (Fig. 1). Additionally, an annual, full-scale survey covering major parts of the Norwegian Sea and adjacent waters has been done each year since the beginning of the millennium, using one or two IMR ships with participating research vessels from Iceland and the Faroe Islands covering their territorial waters.

### Dissolved inorganic nutrients (nitrite, nitrate, phosphate, silicate)

After three rinses, each water sample (20 mL) was collected in a polyethylene vial. Up until the turn of the century (1999–2002) most nutrient samples were analysed in real time onboard the research vessels and without a poison or preservative agent. As the research cruise activity expanded, the number of automated analysers could no longer match the number of IMR ships operating simultaneously (see Gundersen *et al*.^2^ for details) and nutrient samples were added 200 µL chloroform to retard biological activity and stored at +4 °C for analysis in the home laboratory, usually within 1–6 weeks. Prior to analysis, the samples were acclimated to room temperature as the evaporating chloroform was evacuated by vacuum. A number of Automated Analyzer (AA) systems have been in use over the years (Table 2), based on methods first described by Bendschneider & Robinson^3^ and Grasshof^4^, with a number of minor adjustments suggested by the manufacturers (Alpkem, Skalar). The AA system measures nitrate, nitrite, phosphate and silicate. Briefly, nitrate in seawater is reduced to nitrite coupled to a diazonium ion and, in the presence of aromatic amines, the resulting blue azo-dye is determined spectrophotometrically at 540 nm. The nitrate concentration is corrected for ambient nitrite (same analytical method as for nitrate, but without cadmium reduction) measured concurrently. Phosphate reacts with molybdate at low pH and the resulting phosphomolybdate is reduced with ascorbic acid to a blue complex measured spectrophotometrically at 810 nm. With the new Skalar AA purchased in 2017 (Table 2) the phosphomolybdate peak is now measured at 880 nm. Silicate (silicic acid) is reacting to molybdate at low pH and the resulting silico-molybdate is reduced by ascorbic acid to a blue dye measured spectrophotometrically at 810 nm.

**Table 2:** Use of analytical instruments (1990–2019) in the Plankton Chemistry Laboratory showing analytical range, precision and accuracy.

Instrument	Lab. reg.	Years	Parameter	Range	Precision (%)	Accuracy (%)
Skalar-hybrid (Skalar Instruments)	Skalar-A	1983–2001	Nitrate	0.5–20 µmol L^−1^	<0.2	<1
			Nitrite	0.05–2.5 µmol L^−1^	<0.2	<1
			Phosphate	0.02–1.5 µmol L^−1^	<1	<2
			Silicate	0.15–20 µmol L^−1^	<0.2	<1
Skalar-hybrid (Skalar Instruments)	Skalar-B	1978–2008	Nitrate	0.5–20 µmol L^−1^	<0.2	<1
			Nitrite	0.05–2.5 µmol L^−1^	<0.2	<1
			Phosphate	0.02–1.5 µmol L^−1^	<1	<2
			Silicate	0.15–20 µmol L^−1^	<0.2	<1
Akpkem (O.I. Analytical)	Alpkem-C	2004–2014	Nitrate	0.5–20 µmol L^−1^	<0.2	<1
			Nitrite	0.05–3 µmol L^−1^	<0.2	<1
			Phosphate	0.06–3 µmol L^−1^	<2	<2
			Silicate	0.4–20 µmol L^−1^	<0.2	<1
Skalar-hybrid (Skalar Instruments)	Skalar-D	2008–2017	Nitrate	0.4–20 µmol L^−1^	<0.2	<1
			Nitrite	0.06–3 µmol L^−1^	<0.2	<1
			Phosphate	0.06–3 µmol L^−1^	<2	<2
			Silicate	0.7–20 µmol L^−1^	<0.2	<1
Skalar San++ Continuous Flow Analyzer	Skalar-F	2017-present	Nitrate	0.5–50 µmol L^−1^	<0.2	<1
			Nitrite	0.06–5 µmol L^−1^	<0.2	<1
			Phosphate	0.06–5 µmol L^−1^	<1	<2
			Silicate	0.7–150 µmol L^−1^	<0.2	<1
Turner Design 10 AU	Turner	1990-present	ChlA	0.005–0.25 mg m^−3^	<1	<3
			Phaeo	0.005–0.25 mg m^−3^	<1	<3
Carlo-Erba 1106 Strumentazione	Carlo-Erba	1990–2003	POC	0.004–0.7 mg	> 1	<1
			PN	0.001–0.12 mg	<1.5	<1
Thermo Finnegan Flash EA1112	Finnegan	2004–2016	POC	0.004–0.7 mg	<1	<1
			PN	0.001–0.12 mg	<1	<1
rapid MICRO N cube(*)	Elemental	2017-present	POC	0.004–1.2 mg	<1	<1
			PN	0.001–0.2 mg	<1	<1
916 Ti-Touch	Winkler-DO	2019-present	DOW	0.06–90 mL L^−1^	<0.002	NA
916 Ti-Touch	Winkler-DO	2020-present	DOW	0.06–90 mL L^−1^	<0.002	NA
Titrino 665	Dozimat	2000-present	DOW	0.08–90 mL L^−1^	<0.002	NA

(*) = originally designed for N-analysis only; in 2020 the intrument was retrofitted to inlcude C-analysis and renamed *UNICUBE trace*.

Analyzed ChlA and Phaeo shows measuring range in acetone extracted solution, and POC and PN shows analytical range in a filter-collected sample.

### Chlorophyll-a and Phaeopigment samples (ChlA, Phaeo)

A standard volume (265 mL) is collected from each depth (Table 1), filtered onto a 25 mm GFF membrane and stored frozen at −20 °C until analysis in the land-based laboratory. Historically, pigment samples were transported home by one of the cruise participants, as hand-luggage in a cooler with frozen cooler-elements. These days, pigment samples are brought back in specially designed coolers, with an internal temperature recorder, that is rated for −20 °C for a minimum of 3 days. In the laboratory, the samples are thawed in 90% acetone, and stored at +4 °C overnight before analysis on a Turner Design 10 AU fluorometer. Phaeopigments (Phaeo) are measured separately from ChlA, in a second reading of the sample after adding 3 drops of a weak acid (5% HCl). The fluorometer is standardized regularly using a solid standard with known fluorescence, and in accordance with Holm-Hansen & Riemann^5^ and the manufacturer^6^. Up until 2008, drift in the light-source was monitored annually, but from 2009 the solid standard has been recorded every time the fluorometer is used.

### Particulate organic carbon and nitrogen (POC, PN) samples

A standard volume (265 mL) is collected from each depth (Table 1) and filtered onto a pre-combusted 25 mm GFF membrane (+450 °C, min. 4 h). Each sample is stored frozen at −20 °C in a pre-combusted glass tube until analysis in the land-based laboratory. Preparations of samples and analysis of elemental C and N is described in detail in Grasshof *et al*.^7^. Briefly, the dried filter-samples are fumed in acid (conc. HCl) in a desiccator for 4–12 h, before they are dried again and packed in a tin-foil capsule. Analysis of POC and PN is performed on an elemental analyzer (see Table 2 for details) and in accordance with the manufacturer’s recommendations.

### Dissolved oxygen samples by Winkler titrations (DOW)

Samples for dissolved oxygen are collected in volume calibrated glass BOD bottles (approximate volume 125 mL) and filled from bottom up using a Tygon-tubing. The sample is let to overflow approximately 3 times the volume and great care is taken to avoid small air bubbles at the inside of the sample bottle. Thiosulfate titrations of dissolved oxygen are still done as first described by Winkler^8^ but the method has seen some updates and improvements over the years^9–11^. Grasshof *et al*.^7^ is describing in detail the current method of sample collection, pre-treatment, and titrations of Winkler samples. Briefly, dissolved oxygen reacts with an alkaline solution (Reagent 1) to form a manganese-hydroxy-complex. Under alkaline conditions, the Mn-complex is reacting with the iodide solution (Reagent 2) and let to precipitate at the bottom of the sample bottle. The sample is added 10 N sulphuric acid to dissolve the iodide precipitate (pH = 1–2.5) and the yellow iodine is titrated by thiosulfate to a clear solution. The titrant is standardized by a known concentration of potassium iodate (KIO3) as described by Grasshof *et al*.^7^.

### Quality control

All data were quality controlled (QC) by analysts using quality flags 0–5 (Table 3) in accordance with Jaccard *et al*.^12^ and the *OceanSITES Data Format Reference Manual* (http://www.oceansites.org/docs/oceansites_data_format_reference_manual.pdf). Only QC flags 1, 2, and 5 are made available for these data records. “Good data” (flag = 1) are data that passed QC, “unexpected data” (flag = 2) are data that appear not to conform to expected value (see Technical Validation below for details) but we have no reason to exclude them; “corrected data” (flag = 5) are obvious errors based on notes from the sample sheet, where analytically correct data has been relocated to another depth (e.g. where the entire nutrient profile has been mislabelled and logged in “up-side-down”). In cases where single samples appear mislabelled, exhibit analytical errors, or appear to fall outside expected QC envelope, such values are discarded as “bad data” (flags = 3 and 4 are dedicated to doubtful and erroneous data, respectively).

**Table 3:** Quality control (QC) flags used for dissolved inorganic nutrients (nitrite, nitrate, phosphate, silicate), pigments (ChlA, Phaeo), particulates (POC, PN) and Winkler dissolved oxygen (DOW) samples.

ASCII flag	netCDF flag	Action
0	[48, or 48b]	No QC performed
1	[49, or 49b]	Good data
2	[50, or 50b]	Data did not conform to expected outcome, but cannot be discarded
3	[51, or 51b]	Data appears compromised and cannot be corrected
4	[52, or 52b]	Bad data clearly beyond correction
5	[53, or 53b]	Good data that appears misplaced, and has been corrected
6	[54, or 54b]	Data value below detection
7	[55, or 55b]	Measured value beyond detection
8	[56, or 56b]	Interpolated value
9	[57, or 57b]	Missing data
A	[65, or 65b]	Uncertainty of the data value

The dataset is distributed in two file formats; a comma separated values (ASCII format) or a network common data form (NetCDF). Selected data will therefore appear in the ASCII format with numeral flags (ASCII flag), or as 8-byte numerals in the netCDF format (netCDF flag). See Jaccard *et al*.^12^ for more detailed descriptions of the terms used below. The data header will list all the flags listed, but only QC flags 0–5 (ASCII) and flags 48–53 or 48b–53b (netCDF) were used to evaluate our data.

## Data Records

Any use of this 1990–2019 compilation of data should be accompanied by a cititaion of this paper, including a proper use of doi-reference (10.21335/NMDC-482758181) and citation of the actual data^13^. The data are available from the Norwegian Marine Data Center (NMDC) home page at IMR (http://metadata.nmdc.no/metadata-api/landingpage/49ce6ca612889a957aed47019f4a49f3). All data (Table 4) are hosted on an OPeNDAP server at IMR and available both as netCDF4 and csv files, including a pdf-link with reference to a brief description of metadata and methods. The netCDF file (Table 5) follow the SeaDataNet format for profile data (https://www.seadatanet.org/Standards/Data-Transport-Formats). SeaDataNet (https://www.seadatanet.org/) is the standard publication format for biogeochemical data at IMR and the specifics of the netCDF data file format can be found in chapter 4.5.1 of the current version (v.1.22) at https://archimer.ifremer.fr/doc/00454/56547/. Key features of SeaDataNet include rigorous use of the Natural Environment Research Council (NERC, UK) libraries (Table 4), including measurement techniques (P01) and use of data units (P06). The file format is self-contained for both datasets and variables, and we have chosen variable names most familiar to the ocean science community (Table 4). All data in this paper are considered public domain and, as such, some of them (chlorophyll, nitrate, phosphate, silicate, and dissolved oxygen) are also submitted as a minor part of the global data sets in the Copernicus Marine Data Store (https://data.marine.copernicus.eu/products). You will have to sign up with the Copernicus Marine Data Service to get access to the Global Ocean - Delayed Mode Biogeochemical product^14^ for the November 2022 release of data.

**Table 4:** Groups of parameters as they appear in netCDF with method descriptions (P01 strings) as listed by the National Environmental Research Council, the NERC Vocabulary Server (NVS) hosted by the British Oceanographic Data Centre BODC (http://vocab.nerc.ac.uk/search_nvs/).

Parameter	This study	BODC concept ID	Parameter description	Approved BODC units		CMEMS	Norwegian Water Frame Directive	
	Acronym	SDN:P01		SDN:P06	SDN:P06	Acronym	Parameter (Norw.)	Acronym (Norw.)
Nitrate	Nitrate	CHEMM012	Concentration of nitrate per unit volume of water (dissolved plus reactive particulate phase) with a correction for nitrite	UPOX	µmol/L	NTRA	Nitrat	N-NO3
Nitrite	Nitrite	NTRIAAZX	Concentration of nitrite per unit volume of water (unknown phase)	UPOX	µmol/L	NTRI	Nitritt	N-NO2
Phosphate	Phosphate	PHOSAATX	Concentration of phosphate per unit volume of water (dissolved plus reactive particulate phase)	UPOX	µmol/L	PHOS	Fosfat	P-PO4
Silicate	Silicate	SLCAAATX	Concentration of silicate per unit volume of water (dissolved plus reactive particulate phase)	UPOX	µmol/L	SLCA	Silikat	SI-SIO2
Chlorophyll-a	ChlA	CPHLFLP1	Concentration of chlorophyll-a per unit volume of water (particulate > GF/F phase)	UMMC	mg/m3	CPHL	Klorofyll-a	KLFA
Phaeopigments	Phaeo	PHAEFLP1	Concentration of phaeopigments per unit volume of water (particulate > GF/F phase)	UMMC	mg/m3	PHAEO	Feopigmenter	Feo
Particulate organic C	POC	CORGCAP1	Concentration of organic carbon per unit volume of water (particulate > GF/F phase)	UPOX	µmol/L	POC	Partikulært organisk karbon	POC
Particulate N	PN	NTOTCAP1	Concentration of total nitrogen per unit volume of water (particulate > GF/F phase)	UPOX	µmol/L	PN	Partikulært organisk nitrogen	PON
Winkler dissolved oxygen	DOW	DOXYWITX	Concentration of oxygen per unit volume of the water body [dissolved plus reactive particulate phase] by Winkler titration	UMLL	mL/L	DOX1	Winkler oksygen	O2

Associated units (P06) and abbreviations applied for this data set are shown, including acronyms used by the Copernicus Marine Service (CMEMS) and the Norwegian Water Frame Directive (Vanndirektivet).

**Table 5:** Example of netCDF file extracted from SeaDataNet (SDN), showing cruise number (SDN_CRUISE), sampling station (SDN_STATION), measured bottom depth using an ecco sounder (SDN_BOT_DEPTH), and position (Latitude, Longitude).

Time	SDN_CRUISE	SDN_STATION	SDN_BOT_DEPTH	Latitude	Longitude	PRES	depth	Nitrate	Nitrite	Phosphate	Silicate	ChlA	Phaeo	POC	PN	DOW
UTC			m	degrees_north	degrees_east	dbar	m	umol L-1	umol L-1	umol L-1	umol L-1	mg m-3	mg m-3	umol L-1	umol L-1	mL L-1
1993-05-04T05:00:00Z	1993005	361	1750	75.00	7.00	0	0	NaN	NaN	NaN	NaN	0.27	0.16	NaN	NaN	NaN
1993-05-04T05:00:00Z	1993005	361	1750	75.00	7.00	5	5	12.5	0.05	0.825	5.6	0.16	0.09	3.171	0.349	7.92
1993-05-04T05:00:00Z	1993005	361	1750	75.00	7.00	10	10	12.5	0.05	0.865	5.6	0.23	0.15	3.651	0.510	7.82
1993-05-04T05:00:00Z	1993005	361	1750	75.00	7.00	30	30	12.7	0.11	NaN	5.7	0.25	0.16	NaN	NaN	7.80
1993-05-04T05:00:00Z	1993005	361	1750	75.00	7.00	50	50	12.6	0.05	0.855	5.7	0.18	0.10	NaN	NaN	7.35
1993-05-04T05:00:00Z	1993005	361	1750	75.00	7.00	75	75	12.6	0.05	0.835	5.6	0.25	0.19	NaN	NaN	7.22
1993-05-04T05:00:00Z	1993005	361	1750	75.00	7.00	100	100	12.6	0.05	0.845	5.6	0.17	0.11	NaN	NaN	7.31
1993-05-04T05:00:00Z	1993005	361	1750	75.00	7.00	125	125	12.6	0.05	0.865	5.6	NaN	NaN	NaN	NaN	7.32
1993-05-04T05:00:00Z	1993005	361	1750	75.00	7.00	150	150	12.6	0.05	0.875	5.7	NaN	NaN	NaN	NaN	7.32
1993-05-04T05:00:00Z	1993005	361	1750	75.00	7.00	200	200	12.9	0.03	0.875	5.9	NaN	NaN	4.394	0.434	7.35
1993-05-04T05:00:00Z	1993005	361	1750	75.00	7.00	250	250	12.2	0.06	0.905	5.6	NaN	NaN	NaN	NaN	7.74
1993-05-04T05:00:00Z	1993005	361	1750	75.00	7.00	300	300	12.1	0.04	0.845	5.6	NaN	NaN	3.410	0.392	7.76
1993-05-04T05:00:00Z	1993005	361	1750	75.00	7.00	400	400	12.5	0.02	0.845	5.8	NaN	NaN	NaN	NaN	NaN
1993-05-04T05:00:00Z	1993005	361	1750	75.00	7.00	500	500	13.0	0.00	0.895	6.3	NaN	NaN	2.452	0.239	NaN
1993-05-04T05:00:00Z	1993005	361	1750	75.00	7.00	600	600	14.3	0.00	0.985	7.4	NaN	NaN	NaN	NaN	7.10
1993-05-04T05:00:00Z	1993005	361	1750	75.00	7.00	700	700	14.2	0.00	0.975	7.7	NaN	NaN	1.949	0.214	7.20
1993-05-04T05:00:00Z	1993005	361	1750	75.00	7.00	800	800	14.2	0.00	1.005	8.0	NaN	NaN	NaN	NaN	7.20
1993-05-04T05:00:00Z	1993005	361	1750	75.00	7.00	900	900	14.0	0.00	0.995	7.9	NaN	NaN	NaN	NaN	7.19
1993-05-04T05:00:00Z	1993005	361	1750	75.00	7.00	1100	1100	14.8	0.00	1.035	9.5	NaN	NaN	0.002	0.000	7.09
1993-05-04T05:00:00Z	1993005	361	1750	75.00	7.00	1340	1340	15.0	0.00	1.045	10.4	NaN	NaN	0.001	0.000	7.08
1993-05-04T05:00:00Z	1993005	361	1750	75.00	7.00	1500	1500	15.0	0.00	1.055	10.7	NaN	NaN	NaN	NaN	7.12
1993-05-04T05:00:00Z	1993005	361	1750	75.00	7.00	1600	1600	14.9	0.00	1.055	10.6	NaN	NaN	2.775	0.235	7.11
1993-05-04T05:00:00Z	1993005	361	1750	75.00	7.00	1709	1709	15.1	0.00	1.065	10.9	NaN	NaN	NaN	NaN	7.13

Sampling depths are displayed as original pressure readings at the time of collection (PRES) and recalculated to meters (depth) using CTD temperature and density. Dissolved inorganic nutrients (µmol L^−1^ of Nitrate, Nitrite, Phosphate, Silicate), pigment content (mg m^−3^ of ChlA, Phaeo), particulate organic C and N (µmol L^−1^ of POC, PN) and Winkler titrations of dissolved oxygen (mL L^−1^ of DOW), are shown where samples have been collected. Depths without sample collections are denominated ‘not a number’ (NaN). For brevity, columns with quality control (QC) flags, associated with each measured parameter (parameter_SEADATANET_QC) are not included in this overview.

**Fig. 2:**
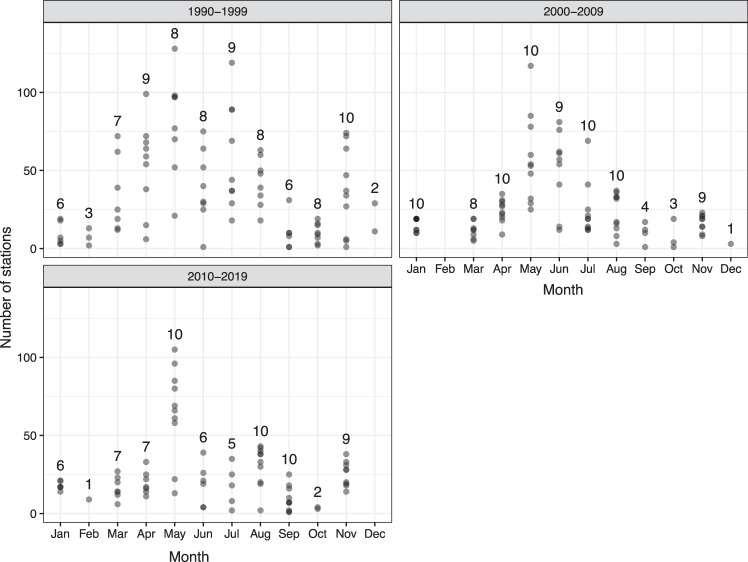
Number of stations visited over the course of a year, during three decades in the Norwegian, Greenland and Iceland Seas (1990–2019). The number above each column (n) shows the number of years sampled during that month, within that decade. Months with the same numbers of stations sampled each year (black dots) are also shown.

**Fig. 3:**
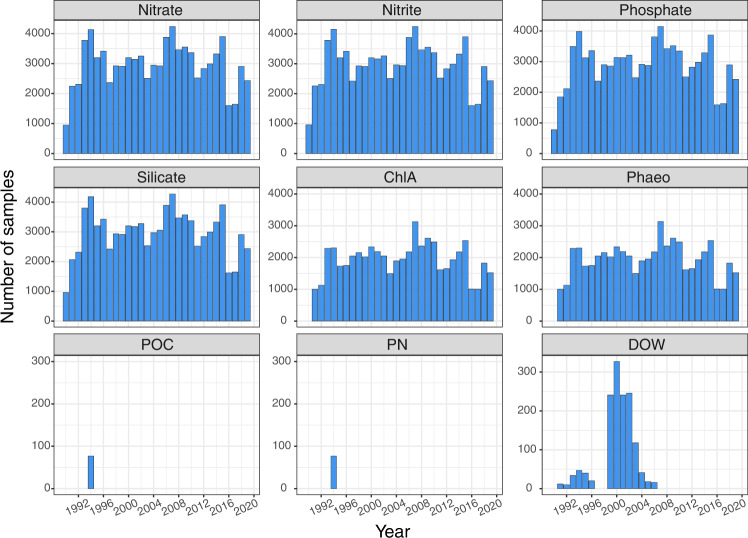
Number of samples collected each year, from the Norwegian, Greenland and Iceland Seas. Note the different scale on the y-axis for particulate elements (POC, PN) and dissolved oxygen (DOW).

Distribution and number of stations visited has changed over the three decades presented here (Fig. 1). The first decade (1990–1999) had sampling stations more scattered over the entire Nordic Seas region and the highest number of stations sampled (total number = 3252). The following two decades saw fewer stations sampled (n = 2230 stations in 2000–2009 and n = 1970 stations in 2010–2019) only covering the Norwegian and Greenland Seas (Fig. 1). Distribution of stations sampled within a year (Fig. 2) shows a maximum coverage in May during all three decades. Each month is not always visited each year (n < 10 in Fig. 2) but April-June, the period of the *Ecosystem Survey*, shows most stations visited in each of the three decades. This data set contains dissolved inorganic nutrient measurements (nitrite, nitrate, phosphate and silicate) and pigments (ChlA and Phaeo) from most sample stations Fig. 3). On some cruises, dissolved oxygen measured by Winkler titrations (DOW) and particulate carbon and nitrogen (POC, PN) were filter-collected between 1990 and 2019 (Fig. 3).

## Technical Validation

Quality control of large scale, long-term data sets are crucial to account for potential mislabeling, potential errors in storage and handling of the samples leading to contamination, and potential anomalies during analysis of the samples. The Plankton Chemistry Laboratory is currently using a QC-flagging system to account for the quality of the data that is produced (Table 3). Data with flags 1, 2 and 5 are made available for use in this publication, while flags 3 and 4 are deemed compromised and are not included. Due to the many different people participating in the sampling program on IMR cruises, we must accept that minor mistakes can be made. Some of these can be corrected (e.g. a mislabeled depths with a value that clearly belongs somewhere else) and are given Flag = 5, while others are beyond correction (e.g. samples that shows signs of contamination or appears to have been stored too long) and are given flags 3 or 4. An important QC flag is number 2, where we label data points that are outside expected value, but for no apparent reason. Unfortunately, data from samples collected prior to 2010 in our time-series used a different QC definition of flag number 2, and these data were excluded. Therefore, flags number 2, 3 and 4 were routinely excluded from data sets during the first two decades (1990–2009).

Our collection of unfiltered seawater samples, added small aliquots of chloroform, is not commonplace in ocean research. However, we have not detected any difference between filtered and unfiltered nutrient samples (see Gundersen *et al*.^2^ for details). Filter-collection and storage of frozen particulate samples (ChlA, Phaeo, POC, PN), and onboard Winkler oxygen titrations (DOW), do follow internationally recognized guidelines for time-series measurements (e.g.^11^). The Plankton Chemistry Laboratory at IMR maintain quality control of precision and accuracy by daily assessments of analytical standard curves of internal standards. Our laboratory find it crucial to maintain contacts with others laboratories through regional intercalibration studies such as QUASIMEME (http://www.quasimeme.org/) and in global intercalibrations such as the International Ocean Carbon Coordination Project (IOCCP) and the Japan Agency for Marine-Earth Science and Technology (JAMSTEC) (https://repository.oceanbestpractices.org/handle/11329/883).

**Fig. 4:**
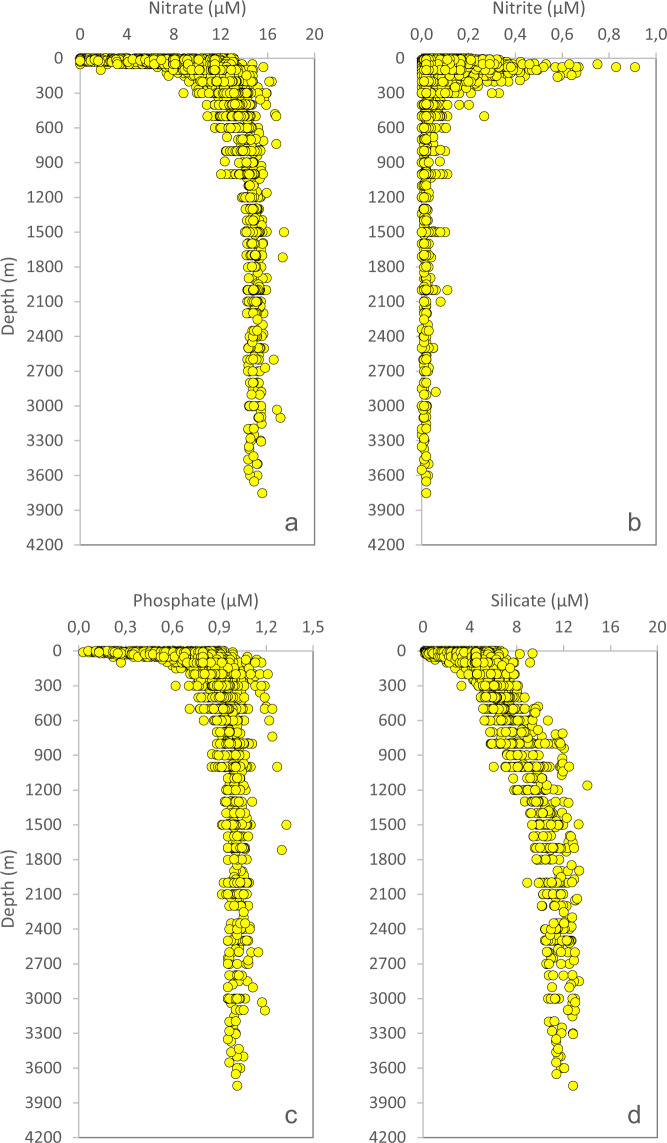
Depth-distribution of nitrate (**a**), nitrite (**b**), phosphate (**c**) and silicate (**d**), of all samples collected in 1993.

**Fig. 5:**
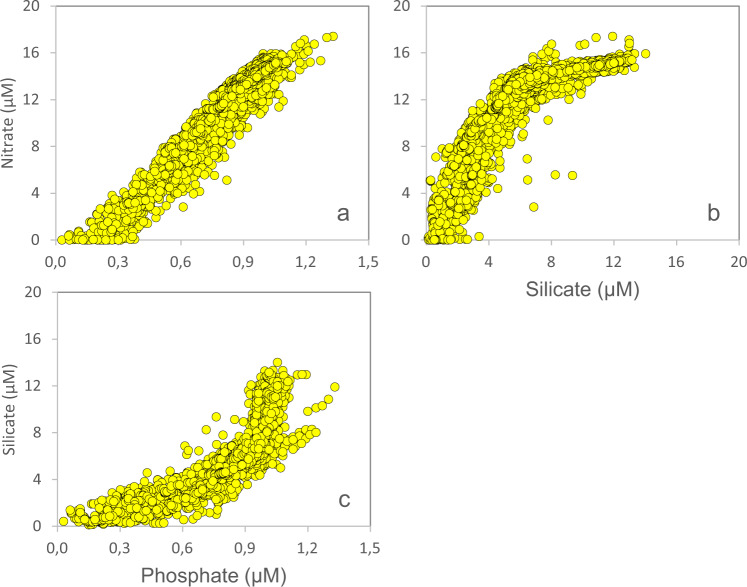
Nitrate concentrations plotted as a function of phosphate (**a**) and silicate (**b**), and silicate concentrations plotted as a function of phosphate (**c**), of all samples collected in 1993.

**Fig. 6:**
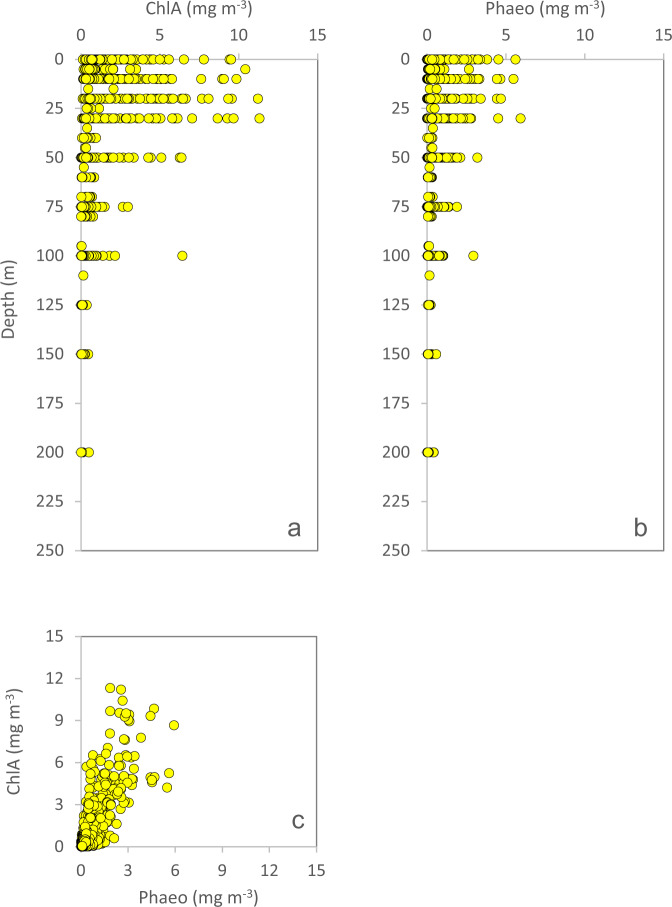
Depth distribution of ChlA (**a**) and Phaeo (**b**), and ChlA plotted as a function of Phaeo (**c**), of all samples collected in 1993.

**Fig. 7:**
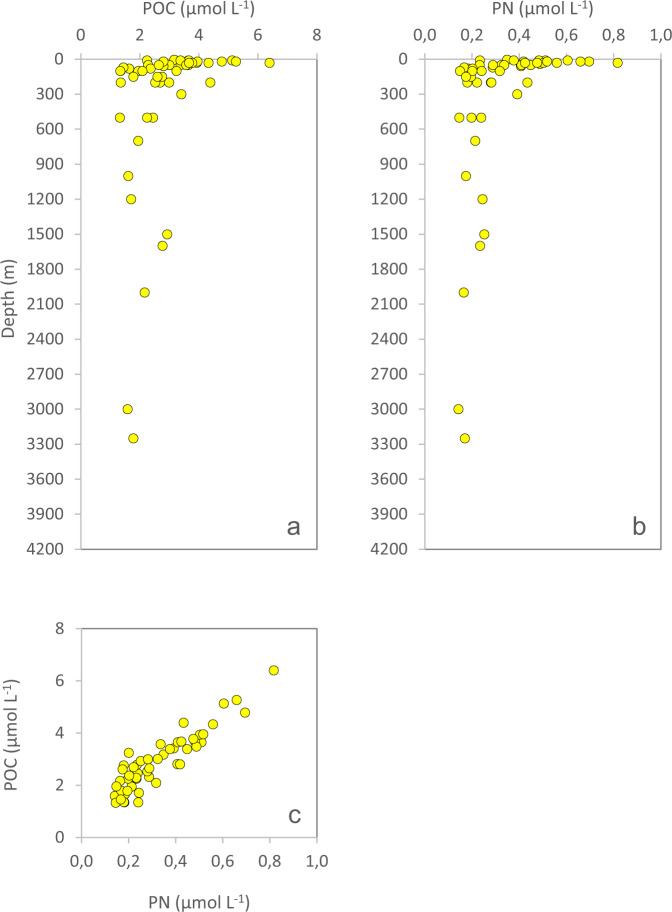
Depth distribution of POC (**a**) and PN (**b**), and POC plotted as a function of PN (**c**), of all samples collected in 1993.

**Fig. 8:**
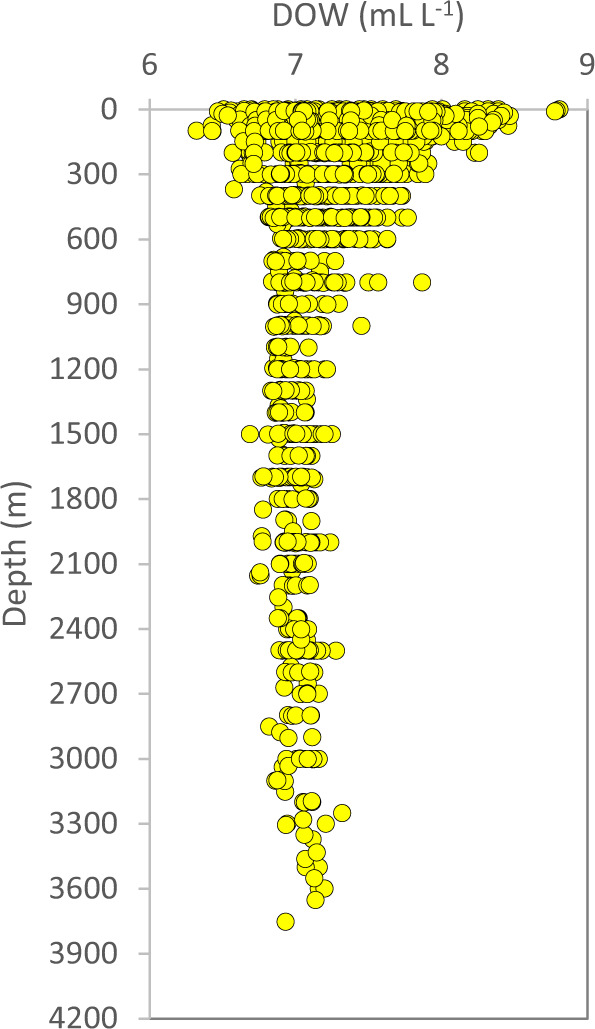
Measured DOW plotted as a function of depth, of all samples collected in 1993.

The laboratory is using a specific set of QC-criteria (details below) and outliers will be appropriately flagged (Table 3). All depth-profiles of nutrients are expected to fall within a “QC-envelope” as a function of season (Fig. 4a–d). We also expect a certain molar relationship between most of the macronutrients (Fig. 5a–c). The filter-collected pigments ChlA and Phaeo (Fig. 6a,b), the particulates POC and PN (Fig. 7a,b) and DOW concentrations (Fig. 8) also have an expected depth profile, but with a high seasonal variability in surface waters. Therefore, a wide range of values are expected in surface waters, depending on time of the year and nutrient availability. As the macronutrients nitrate, phosphate and silicate disappear, a seasonal increase in phytoplankton biomass (ChlA, Phaeo, POC, PN) is expected in surface waters. Therefore, low nutrient concentrations in surface waters are most often associated with elevated concentrations of ChlA, Phaeo, POC and PN. We also expect a semi-constant internal relationship between the two pigments (Fig. 6c) as we are for the particulate elements (Fig. 7c). Elevated rates of photosynthesis in spring-summer will cause a net increase in DOW concentrations (Fig. 8). Extracellular byproducts from photosynthesis include dissolved organic nitrogen, which is subject to decay into ammonium and further bacterial nitrification into nitrite. Therefore, as macronutrients are approaching a minimum in spring-summer, we usually observe an increase in nitrite concentrations in surface waters (Fig. 4b). Prior to flagging of a parameter, a potential outlier will have to stand out in two or more of the QC-plots indicated above. Finally, operators performing the QC-assessments are mindful to avoid “data grooming” (i.e. applying a too strict QC-envelope), as we may then miss minor changes in the data set that could appear significant on longer term (e.g. decadal) temporal scale.

## Data Availability

No custom code was used to generate or process the data described in this paper.

## References

[1] Skjoldal, H. R. *Background – “Mare cognitum” and this book* (ed. Skjoldal, H. R.) *The Norwegian Sea ecosystem* (Tapir Academic Press, 2004).

[2] Gundersen, K. *et al*. Thirty years of nutrient biogeochemistry in the Barents Sea and the adjoining Arctic Ocean, 1990 – 2019. *Sci. Data*10.1038/s41597-022-01781-w (2022). 10.1038/s41597-022-01781-w PMC9588074 36273001

[3] Bendschneider K, Robinson RI (1952). A new spectrophotometric method for the determination of nitrite in seawater. J. Mar. Res..

[4] Grasshoff, K. On the automatic determination of phosphate, silicate and fluoride in seawater (ICES Hydrographic Committee Report No. 129, 1965).

[5] Holm-Hansen O, Riemann B (1978). Chlorophyll a determination: improvements in methodology. Oikos.

[6] Turner Designs Model 10-AU-005 Field Fluorometer User’s Manual, Version S1C (Turner Designs, California, USA, 1992).

[7] Grasshoff, K., Ehrhart, M. & Kremling, F. *Methods of seawater analysis 2nd edn* (Verlag Chemie, Wiley, Weinheim, 1983).

[8] Winkler LW (1888). Die Bestimmung des im wasser gelösten Sauerstoffes. Ber. Dtsch. Chem. Ges..

[9] Carpenter JH (1965). The Chesapeake Bay Institute. Technique for the Winkler oxygen method. Limnol. Oceanogr..

[10] Murray JN, Riley JP, Wilson TRS (1968). The solubility of oxygen in Winkler reagents used for the determination of dissolved oxygen. Deep-Sea Res..

[11] Strickland, J.D.H & Parsons, T.R. *A Practical Handbook of Seawater Analysis* (Fisheries Research Board of Canada, Bulletin **167**, (1972).

[12] Jaccard, P. *et al*. Quality information document for global ocean reprocessed *in-situ* observations of biogeochemical products (Issue 2.0) 10.13155/54846 (2018).

[13] Gundersen K (2022). Institute of Marine Research.

[14] Copernicus Marine *in situ* TAC. Copernicus Marine *In Situ* - Global Ocean - Delayed Mode Biogeochemical product 10.17882/86207 (2023).

